# Development of Secondary Cancers in Pre- vs Post-ICI Eligibility Periods for Metastatic Cancers

**DOI:** 10.1001/jamanetworkopen.2025.57807

**Published:** 2026-02-04

**Authors:** Athena Li, Jiyeong Kim, Thet Su Win, Michael L. Chen

**Affiliations:** 1 Center for Digital Health, Stanford University School of Medicine, Stanford, California; 2 Department of Dermatology, Brigham and Women’s Hospital, Boston, Massachusetts; 3 The University of Texas at Austin Dell Medical School; 4 Department of Dermatology, Stanford University School of Medicine, Stanford, California

## Abstract

This cohort study investigates incidence of secondary primary cancers before vs after approval of immune checkpoint inhibitors (ICIs) among patients with metastatic cancers.

## Introduction

Immune checkpoint inhibitors (ICIs) have become first-line treatment for advanced malignant neoplasms.^1,2^ With improved survival outcomes due to ICIs, treatment-related sequelae are increasingly observed.^3,4^ Although the risk of developing secondary primary cancers (SPCs) has been preliminarily studied for metastatic melanoma, the risk is not well understood for metastatic cancers in general despite ICIs’ expanded use.^5^ We conducted a cohort analysis to determine whether SPC incidence changed after the Food and Drug Administration (FDA) approved ICIs for 4 metastatic malignant neoplasms, including cutaneous melanoma (CM), non–small cell lung cancer (NSCLC), hepatocellular carcinoma (HCC), and urothelial carcinoma (UC).

## Methods

This cohort study followed the STROBE reporting guideline. In accordance with the Common Rule, this study was exempt from review and informed consent due to use of publicly deidentified data. We selected patients with a first primary diagnosis of 1 of 4 metastatic malignant neoplasms for which ICIs are first-line treatment (CM, NSCLC, HCC, and UC) from the Surveillance, Epidemiology, and End Results database (SEER 17; 2004-2021). We identified metastatic cancers based on the corresponding American Joint Committee on Cancer 6th, 7th, or 8th edition code. We calculated standardized incidence ratios (SIRs) as the number of SPCs among cancer cases relative to expected primary cancer incidence rates in the US general population adjusted for age, sex, and race and ethnicity (eMethods 1 in Supplement 1). SIRs accounted for differing lengths of follow-up for each cancer and period. We defined pre- and post-ICI periods based on year of FDA approval for each malignancy (eMethods 2 in Supplement 1). SIRs and 95% CIs were compared before and after FDA approval of ICIs. Data were analyzed from May 2024 through November 2025. Statistical significance was determined at *P* = .05 with 2-sided tests using Python programming language version 3.12.7 (Python Software Foundation).

## Results

Among 185 755 patients with a primary metastatic cancer diagnosis from 2004 to 2021 (median [IQR] age at diagnosis, 67 [57-67] years; 44.0% women), we identified 3885 patients (2.1%) diagnosed with an SPC. Patient demographics were similar across pre-ICI and post-ICI groups (Table 1).

**Table 1.** Patient Characteristics

Characteristic	Patients, No. (%) (N = 185 755)							
	CM		NSCLC		HCC		UC	
	Pre-ICI (n = 2747)	Post-ICI (n = 5559)	Pre-ICI (n = 110 302)	Post-ICI (n = 53 315)	Pre-ICI (n = 5575)	Post-ICI (n = 2554)	Pre-ICI (n = 3787)	Post-ICI (n = 1916)
Period	2004-2010	2011-2021	2004-2015	2016-2021	2004-2016	2017-2021	2004-2016	2017-2021
Sex								
Male	1863 (67.8)	3839 (69.1)	59 928 (54.3)	27 741 (52.0)	4555 (81.7)	2077 (81.3)	2707 (71.5)	1366 (71.3)
Female	884 (32.2)	1720 (30.9)	50 374 (45.7)	25 574 (48.0)	1020 (18.3)	477 (18.7)	1080 (28.5)	550 (28.7)
Race and ethnicity^a^								
Hispanic	159 (5.8)	381 (6.9)	7592 (6.9)	4738 (8.9)	1102 (19.8)	549 (21.5)	331 (8.7)	181 (9.5)
Non-Hispanic American Indian or Alaska Native	8 (0.3)	22 (0.4)	402 (0.4)	239 (0.5)	52 (0.9)	26 (1.0)	11 (0.3)	10 (0.5)
Non-Hispanic Asian or Pacific Islander	47 (1.7)	103 (1.9)	9455 (8.6)	6644 (12.5)	888 (15.9)	338 (13.2)	163 (4.3)	118 (6.2)
Non-Hispanic Black	45 (1.6)	85 (1.5)	13 340 (12.1)	6544 (12.3)	824 (14.8)	378 (14.8)	352 (9.3)	153 (8.0)
Non-Hispanic White	2482 (90.4)	4953 (89.1)	79 437 (72.0)	35 003 (65.7)	2702 (48.5)	1254 (49.1)	2928 (77.3)	1452 (75.8)
Non-Hispanic unknown	6 (0.2)	15 (0.3)	76 (0.1)	147 (0.3)	7 (0.1)	9 (0.4)	2 (0.1)	2 (0.1)
Age, y								
≤54	911 (33.2)	1419 (25.5)	17 626 (16.0)	5847 (11.0)	1246 (22.4)	287 (11.2)	445 (11.8)	167 (8.7)
55-69	986 (35.9)	2222 (40.0)	48 768 (44.2)	24 090 (45.2)	2907 (52.1)	1491 (58.4)	1475 (39.0)	791 (41.3)
≥70	850 (30.9)	1918 (34.5)	43 908 (39.8)	23 378 (43.9)	1422 (25.5)	776 (30.4)	1867 (49.3)	958 (50.0)

Abbreviations: CM, cutaneous melanoma; HCC, hepatocellular carcinoma; ICI, immune checkpoint inhibitor; NSCLC, non–small cell lung cancer; UC, urothelial carcinoma.

^a^ Race and ethnicity groups were determined by Surveillance, Epidemiology, and End Results (SEER) database classifications and included Hispanic, non-Hispanic American Indian or Alaska Native, non-Hispanic Asian or Pacific Islander, non-Hispanic Black, non-Hispanic White, and non-Hispanic unknown. Race and ethnicity were assessed for demographic characterization.

There was no significant difference in the overall incidence of SPCs in pre- vs post-ICI groups across the 4 metastatic cancers (Table 2). Patients who were diagnosed with metastatic CM, NSCLC, or UC were more likely than the US general population to be diagnosed with an SPC regardless of their ICI group (Table 2). The cancer with the highest incidence of SPCs was UC in pre-ICI (SIR, 2.64; 95% CI, 2.29-3.04) and post-ICI (SIR, 2.96; 95% CI, 2.30-3.74) groups. The next highest incidence of SPCs was for CM in pre-ICI (SIR, 1.50; 95% CI, 1.27-1.77) and post-ICI (SIR, 1.61; 95% CI, 1.41-1.84) groups. Patients diagnosed with metastatic HCC did not have a difference in SPC incidence compared with the general population.

**Table 2.** SIRs for Secondary Primary Cancers for Metastatic Cancers, 2004-2021

Cancer	SIR (95% CI)			Observed cases^a^			Mean person-years at risk^a^	*P* value (pre- vs post-ICI period)
	All	HC	SC	All	HC	SC		
CM								
Pre-ICI period (2004-2010)	1.50 (1.27-1.77)	2.25 (1.37-3.47)	1.43 (1.19-1.70)	146	20	126	2.47	NA^b^
Post-ICI period (2011-2021)	1.61 (1.41-1.84)	1.85 (1.20-2.74)	1.59 (1.38-1.82)	228	25	203	1.78	.51
NSCLC								
Pre-ICI period (2004-2015)	1.23 (1.18-1.28)	0.82 (0.69-0.97)	1.27 (1.21-1.32)	2273	134	2139	1.09	NA^b^
Post-ICI period (2016-2021)	1.16 (1.09-1.24)	0.90 (0.69-1.15)	1.19 (1.11-1.27)	895	63	832	0.98	.13
HCC								
Pre-ICI period (2004-2016)	1.05 (0.79-1.36)	1.09 (0.35-2.54)	1.04 (0.77-1.38)	55	5	50	0.68	NA^b^
Post-ICI period (2017-2021)	0.97 (0.59-1.50)	1.62 (0.33-4.75)	0.91 (0.53-1.45)	20	3	17	0.52	.77
UC								
Pre-ICI period (2004-2016)	2.64 (2.29-3.04)	1.14 (0.49-2.24)	2.80 (2.41-3.22)	198	8	190	1.06	NA^b^
Post-ICI period (2017-2021)	2.96 (2.30-3.74)	2.20 (0.71-5.13)	3.04 (2.34-3.87)	70	5	65	0.69	.43

Abbreviations: CM, cutaneous melanoma; HC, hematopoietic cancer; HCC, hepatocellular carcinoma; ICI, immune checkpoint inhibitor; NA, not applicable; NSCLC, non–small cell lung cancer; SC, solid cancer; SIR, standardized incidence ratio; UC, urothelial carcinoma.

^a^ The observed number of cases and mean person-years at risk are shown within each group.

^b^ Reference group.

## Discussion

In this cohort study, we did not observe significant differences in SPC risk in pre- vs post-ICI periods across 4 cancers, which differs from prior literature investigating melanoma from 2005 to 2016 and primary cancers from 2013 to 2018.^5,6^ We identified an increased risk of SPCs for 3 of 4 metastatic cancers for which ICIs are first-line therapy. These findings suggest that previously reported associations between ICI therapy and SPC risk may reflect shared risk factors rather than treatment-induced immunomodulation. Further research is warranted to inform clinical decision-making and patient counseling regarding risks associated with immunotherapy.

Limitations include, first, the assumption that patients diagnosed after ICI approval were greatly enriched for receipt of immunotherapy compared with patients diagnosed prior to FDA ICI approval. Second, shorter follow-up in post-ICI groups may underrepresent SPC risk, although SIRs adjusted for varied follow-up time. Third, SEER captures only half of patients with cancer in the US. Our findings support increased cancer monitoring after diagnosis of metastatic cancers and suggest that risk of SPCs after a metastatic cancer diagnosis has not increased since FDA approval of ICIs.

## Notes

Supplement 1. **eMethods 1.** Statistical Methods **eMethods 2.** Pre- and Post-ICI Periods by Primary Metastatic Cancer

Supplement 2.
Data Sharing Statement
