# Supplementary material for: Development of Secondary Cancers in Pre- vs Post-ICI Eligibility Periods for Metastatic Cancers
Source: JAMA Netw Open. 2026 Feb 4;9(2):e2557807. doi: 10.1001/jamanetworkopen.2025.57807 (PMC12873763; doi:10.1001/jamanetworkopen.2025.57807)
Supplement: Supplement 1. — eMethods 1. Statistical Methods eMethods 2. Pre- and Post-ICI Periods by Primary Metastatic Cancer [file jamanetwopen-e2557807-s001.pdf]

Supplemental Online Content

Li A, Kim J, Win TS, Chen ML. Development of secondary cancers in pre- vs post-ICI eligibility periods for metastatic cancers. *JAMA Netw Open*. 2026;9(2):e2557807. doi:10.1001/jamanetworkopen.2025.57807

eMethods 1. Statistical Methods
eMethods 2. Pre- and Post-ICI Periods by Primary Metastatic Cancer

This supplemental material has been provided by the authors to give readers additional information about their work.

© 2026 Li A et al. *JAMA Network Open*.

eMethods 1. Statistical Methods.

We computed standardized incidence ratios (SIRs) as the number of second primary cancers among cancer cases relative to the expected primary cancer incidence rate in the U.S. general population as calculated within SEER*Stat. Using SEER*Stat, SIRs were adjusted to the general population for age (5-year intervals through 84 years and one group for 85+ years), sex (male, female), race (White/other unspecified, Black, Other [American Indian, Alaskan Native, Asian/Pacific Islander]), and calendar-year. Follow-up for secondary cancer incidence was from two months after the date of diagnosis (starting in January 2004) through December 2021 (or, if deceased, date of death). Only the first primary cancer after the index cancer was included. Metastatic cancers were defined by the corresponding AJCC edition definition based on the date of diagnosis (AJCC 6th edition, 2004-2015; AJCC 7th edition, 2016-2017; AJCC 8th edition, 2018-2021). Logarithmic SIRs in the pre-ICI and post-ICI eras were compared via two-sample z-tests. We identified and stratified SPCs by hematopoietic or solid secondary tumors. Hematopoietic secondary cancers included Hodgkin lymphoma, non-Hodgkin lymphoma, myeloma, and leukemia. We followed the Strengthening the Reporting of Observational Studies in Epidemiology (STROBE) reporting guidelines. Confidence intervals for SIRs were calculated at a significance level of 0.05.

© 2026 Li A et al. *JAMA Network Open*.

eMethods 2. Pre- and Post-ICI Periods by Primary Metastatic Cancer.

Cancer	Pre-ICI Period	Post-ICI Period
Metastatic Cutaneous Melanoma	2004-2010	2011-2021
Metastatic NSCLC	2004-2015	2016-2021
Metastatic HCC	2004-2016	2017-2021
Metastatic Urothelial Carcinoma	2004-2016	2017-2021

© 2026 Li A et al. *JAMA Network Open*.
