# Supplementary material for: Development of Secondary Cancers in Pre- vs Post-ICI Eligibility Periods for Metastatic Cancers
Source: JAMA Netw Open. 2026 Feb 4;9(2):e2557807. doi: 10.1001/jamanetworkopen.2025.57807 (PMC12873763; doi:10.1001/jamanetworkopen.2025.57807)
Supplement: Supplement 2. — Data Sharing Statement [file jamanetwopen-e2557807-s002.pdf]

## Data Sharing Statement

Li. Development of Secondary Cancers in Pre- vs Post-ICI Eligibility Periods for Metastatic Cancers. *JAMA Netw Open*. Published online February 4, 2026. doi:10.1001/jamanetworkopen.2025.57807

**Data**
**Data available:** No

**Additional Information**
**Explanation for why data not available:** Data are publicly available via the Surveillance, Epidemiology, and End Results Program after completion of the Access Request Form.
